# Sex-Specific Differences in the Toxic Effects of Heavy Fuel Oil on Sea Urchin (*Strongylocentrotus intermedius*)

**DOI:** 10.3390/ijerph18020499

**Published:** 2021-01-09

**Authors:** Xuanbo Wang, Hang Ren, Xishan Li, Huishu Chen, Zhonglei Ju, Deqi Xiong

**Affiliations:** College of Environmental Science and Engineering, Dalian Maritime University, Dalian 116026, China; wangxuanbo@hotmail.com (X.W.); renhang960810@163.com (H.R.); lxsdmu@outlook.com (X.L.); 15942678549@163.com (H.C.); jzl621925@163.com (Z.J.)

**Keywords:** HFO WAFs, acute toxicity, sea urchin, offspring development

## Abstract

The purpose of this study was to explore and compare the sex-specific differences in the toxic effects of water-accommodated fractions of 380# heavy fuel oil (HFO WAF) on the sea urchin *Strongylocentrotus intermedius*. Sea urchins were acutely exposed to HFO WAF at different nominal concentrations (0%, 10% and 20%) for seven days. The results showed that females had a higher polycyclic aromatic hydrocarbons (PAHs) bioaccumulation in gonad tissues and that both the total antioxidant capacity (TAC) and lipid peroxidation (LPO) levels in the gonad tissues of females were much higher than those of males. The PAHs bioaccumulation in gametes indicated that parents’ exposure could lead to a transfer of PAHs to their offspring, and eggs had higher TAC and LPO than sperms. After maternal and paternal exposure to HFO WAF, the frequency of morphological abnormalities of the offspring was increased when compared to the control. Overall, these results indicated that maternal exposure to HFO WAF could cause more significantly toxic effects on sea urchins than paternal exposure could, which could lead to more significantly negative effects on their offspring.

## 1. Introduction

Recently, oil pollution, which is mainly derived from factory sewage and oil spills, has been one of the major concerns in the marine ecological environment. Once an oil spill occurs, the floating oil could cause a decrease in the oxygen content of the seawater and release aromatic hydrocarbons into the water column, leading to huge damage to the marine ecosystem and producing certain toxic effects on marine organisms [1,2]. With their high bioaccumulation and toxicity, polycyclic aromatic hydrocarbons (PAHs) have generally been known as the main components of crude oil to pose primary potential risks to marine organisms [3,4].

Sea urchins, common members of marine benthos, play an important role in the structure and diversity of marine communities [5]. The life history of sea urchins includes two stages: planktonic larvae and benthic adults. Due to being extremely sensitive to pollution [6,7], sea urchins at an early plankton stage have been regarded as an effective model organism for marine ecotoxicology research and environmental monitoring [8,9]. Previous studies have found that exposure to environmental pollutants could affect the life-history traits of sea urchin offspring in the early developmental stage, manifesting as a reduction in the number of offspring larvae entering the next life-history stage [10], and might subsequently lead to a decline in the population. Therefore, evaluating the impact of environmental pollutants on sea urchins has a potential value for planning and decision-making.

Marine filter-feeding animals exposed to petroleum hydrocarbons can accumulate hazardous substances in vivo and produce reactive oxygen species (ROS) [11]. Once the ROS metabolism is imbalanced, this would lead to the increase in the ROS level, which could cause severe oxidative damage of macromolecules (DNA, lipids and proteins) [11,12]. It is generally known that the toxic mechanisms of oil-derived hydrocarbons are mainly related to the imbalance between ROS production and antioxidant defense systems [13,14,15]. For instance, exposure to benzo[a]pyrene inhibited the activities of glutathione S-transferase (GST) in sea urchins [16]. PAH sediments’ exposure increased in the ROS level and total antioxidant capacity (TAC) of sea urchins [17]. However, these studies mainly focused on the effects of petroleum hydrocarbons on the parent generation, and little is known about the impact on the offspring.

In addition, the overproduction of ROS may cause changes in the gonad function of the parent sea urchins, which may have the potential to transgenerationally affect the quality of gametes and the development of offspring. However, most researchers focus on the transgenerational responses caused by the maternal effect of sea urchins [18,19], and there is a lack of research on the paternal effect. Therefore, it is still uncertain whether there are sex-specific differences in the effects of the developmental and oxidative damage in the offspring of sea urchins exposed to petroleum hydrocarbons. In the present study, we exposed sea urchin (*Strongylocentrotus intermedius*) adults to water-accommodated fractions (WAF) of 380# heavy fuel oil (HFO) with different concentrations for seven days, the changes in TAC and lipid damage of the parental sea urchin gonad tissues, gametes and the larvae at 48 h post-fertilization (hpf) were analyzed, the phenotype of offspring was analyzed, and we assessed the transgenerational effects of sex differences in sea urchins.

## 2. Materials and Methods

### 2.1. Preparation of Water-Accommodated Fractions (WAF) of 380# Heavy Fuel Oil (HFO)

HFO 380# was purchased from Dalian Marine Fuel Co., Ltd., China. The natural seawater used for the WAF preparation and sea urchin culture was from Dalian Xinghai Park, with a salinity of 34 parts per thousand (ppt). HFO WAF was prepared according to the method of a previous study, with minor modifications [20]. Briefly, HFO WAF was prepared with the prefiltered natural seawater (salinity: 34 ppt) at an oil-water ratio of 1: 40 (*w*/*v*). The mixture was stirred for 18 h with low mixing energy using a magnetic stirrer and was then settled for 6 h. After settling, the WAF solution was separated from the oil phase and stored at 4 °C in the dark until subsequent treatment tests.

### 2.2. Sea Urchin Maintenance and Toxicity Tests

Adult sea urchins were purchased from Dalian Haibao Fishery Co., Ltd (Dalian, China). To screen the maturity and sex, 0.5 mL of 0.5 M KCl was injected into the coelom of sea urchins according to our previous research [21]. Sea urchin females and males were kept separately in a seawater recirculation system (Huixin Titanium Co. Ltd., Dalian, China) at 16 ± 0.5 °C under a photoperiod of 12: 12 (light: dark) for a 14-days acclimation and were fed kelp *Saccharina japonica* every 3 days. The animal protocols used in this work were evaluated and approved by the Animal Use and Ethic Committee (CEUA) of the Institute Pasteur Montevideo (Protocol 2009_1_3284). They are in accordance with FELASA guidelines and the National law for Laboratory Animal Experimentation (Law no. 18.611).

After the acclimation period, six sea urchins were randomly assigned to six treatments: controls from both sexes were only exposed to 0.45 μM prefiltered natural seawater; females and males were exposed to 10% and 20% WAF for seven days with three replicates each. During the exposure period, the treatment conditions were also set as 16 ± 0.5 °C, with a photoperiod of 12 h light: 12 h dark but without feeding, and treatment solutions were replaced every 24 h. After the seven-day exposure period, 1 mL of 0.5 M KCl was injected into the coelom of adult sea urchins for spawning. After spawning for 30 min, sea urchins were dissected in an ice bath, and the gonad tissues were acquired and immediately stored at −80 °C for subsequent biochemical analyses.

**Figure 1:**
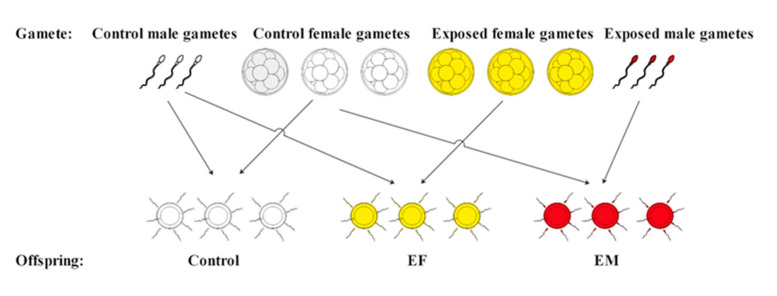
The schematic diagram of the gametes combinatorial design of sea urchins at each oil-loading treatment: control (control female gametes fertilized by control male gametes), EF (exposed female gametes fertilized by control male gametes) and EM (control female gametes fertilized by exposed male gametes).

To further obtain the fertilized eggs, the fertilization procedure was performed based on the method of our previous studies [21,22]. Briefly, 20 μL of sperm were diluted into 50 mL of filtered seawater by a 0.45 μM filter and were then slightly added into 550 mL of an egg suspension solution. To assess the maternal and paternal effects, we set three independent embryo populations with each possible parental cross for different treatments: control female gametes fertilized by control male gametes (Control), exposed female gametes fertilized by control male gametes (EF) and control female gametes fertilized by exposed male gametes (EM) (Figure 1). The fertilized eggs were cultured at 16 ± 0.5 °C for 48 hpf (pluteus stage). The malformation effects were observed and recorded using an inverted microscope (OLYMPUS IX73, Japan). Normal larvae were detected as larvae meeting the following morphological criteria: (1) reached the four-arm pluteus stage developmental endpoint; (2) showed a left/right and abdominally symmetrical body; (3) displayed an excellent differentiated gut at 48 hpf; and (4) showed full skeletal rods and developed arms at 48 hpf. The malformed larvae included abnormal larvae and delayed larvae.

### 2.3. Chemicals Analysis

Water samples (200 mL) were obtained for PAHs measured from the exposure solution every 24 h. Samples were extracted using the EPA 3510C liquid-liquid extraction method [23]. The concentrate was cleaned using the EPA 3630C silica gel cleanup method [24]. Then, PAHs were analyzed by a gas chromatography-coupled mass selective detector.

PAHs in tissue samples were extracted according to a previous study, with modifications [25]. In brief, the sample was placed in a freeze dryer for 24 h and was then ground to a powder with a mortar. The dry weight (DW) of the sample was measured. The sample was transferred to a colorimetric tube, and n-hexane was added in a ratio of 10 mL/g DW. The sample was extracted by ultrasonic for 20 min, and purified anhydrous sodium sulfate was added, after which the extract solution was filtered by a 0.45 μM filter and then concentrated to 1 mL. 4 mL of n-hexane was added to 1 mL of the sample extract solution for extraction, using a turbocharged Vap to concentrate the extract to 1 mL and for the cleanup. The concentrate was cleaned using the EPA 3630C silica gel cleanup method [24]. Then, PAHs were analyzed using a gas chromatography-coupled mass selective detector.

The content of 16 priority PAHs were analyzed, including naphthalene, acenaphthylene, acenaphthene, fluorene, phenanthrene, anthracene, fluoranthene, pyrene, benzo[a]anthracene, chrysene, benzo[b]fluoranthene, benzo[k]fluoranthene, benzo[a]pyrene, indeno[1,2,3-cd]pyrene, dibenz[a,h]anthracene and benzo[ghi]perylene, according to our previous study by gas chromatography-mass spectrometry in selective ion monitoring mode [22]. The recovery of PAHs was between 72% and 118%.

### 2.4. Biochemical Analysis

TAC in gonadal tissue, gametes and larvae of tissues samples was measured by using a total antioxidant capacity assay kit (Nan Jing Jian Cheng Bioengineering Institute, Nanjing, China) based on the colorimetric technique, as described by a previous study [26]. Briefly, the antioxidation substance was used to reduce ferric ion (Fe^3+^) to ferrous ion (Fe^2+^). Fe^2+^ was combined with phenanthrene to form stable complexes, which were measured at 520 nm by ultraviolet spectrometry. The sample was calculated for its antioxidant capacity via the absorbance value and was expressed as U/mg protein.

The malondialdehyde (MDA, a biomarker of membrane lipid peroxidation) content of the tissues samples was measured using the Malondialdehyde assay kit (Nan Jing Jian Cheng Bioengineering Institute, China), based on the thiobarbituric acid method [27]. Briefly, the homogenate supernatant, sample (concentration of 10 nmol/mL tetraethoxypropane) or blank sample (5% glacial acetic acid) was added to the 1% TBA of the same volume in a 40-min water bath at 95 °C. The maximum absorption was measured at 532 nm by using ultraviolet spectrometry, and the calculated MDA content was expressed as nmol/mg protein.

### 2.5. Statistical Analysis

All results were analyzed using SigmaPlot Ver 12.5 (Systat Software, San Jose, CA, USA). PAHs bioaccumulation was tested by a one-way analysis of variance (ANOVA). TAC, LPO levels and the offspring development of the sea urchins were analyzed by a two-way ANOVA. The differences were considered as significant at *p* < 0.05. Statistical analysis data are presented as the mean ± standard deviation (SD).

## 3. Results

### 3.1. Analytical Chemistry

**Figure 2:**
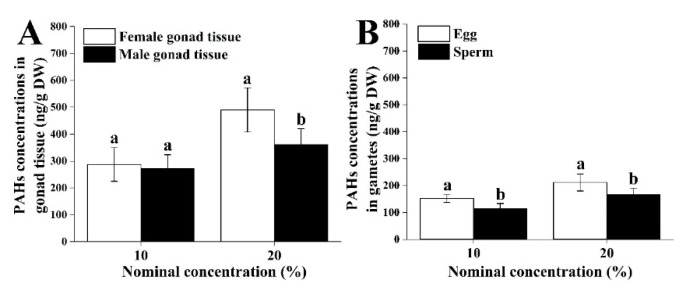
The concentrations of polycyclic aromatic hydrocarbons (PAHs) accumulated in (**A**) gonad tissues and (**B**) gametes from sea urchin females and males exposed to HFO WAFs for seven days. Data were presented as means ± SD. Lowercase letters indicate significant differences between sexes (*p* < 0.05). DW stands for dry weight.

PAHs’ concentration in the water sample was 3.24 μg/L at 10% and 6.38 μg/L at 20%. Sea urchins exposed to a high HFO treatment showed an increased PAHs bioaccumulation in gonad tissues and gametes. Throughout the exposure period, the concentrations of accumulated PAHs in the female gonad tissues (490.05 ± 82.64 ng/g DW) were significantly higher than for the male gonad tissues (361.1 ± 59.06 ng/g DW) at 20% HFO WAF (Figure 2A). There was no significant difference between female and male gonad tissues at 10% HFO WAFs (for females it was 287.12 ± 62.29 ng/g DW, for males it was 271.97 ± 51.54 ng/g DW). PAHs bioaccumulation values were greater in gonad tissues than in gametes. The eggs showed significantly higher concentrations of PAHs than the sperms did (Figure 2B).

### 3.2. Total Antioxidant Capacity Level

**Figure 3:**
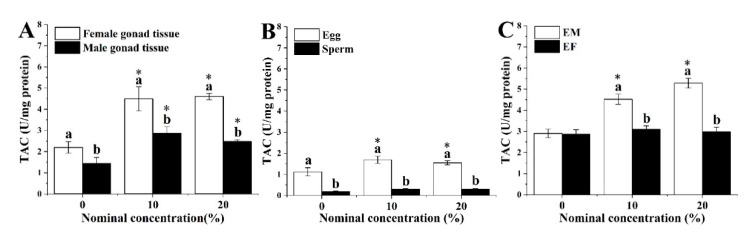
Levels of total antioxidant capacity (TAC) in (**A**) gonad tissues, (**B**) gametes (**C**) and larvae derived from different parental exposed behaviors with HFO WAFs for seven days. Data are presented as means ± SD. Lowercase letters indicate significant differences between sexes. An asterisk (*) labeled above bars indicates significant differences between treatments and the control (*p* < 0.05).

The TAC levels in gonad tissues, gametes and larvae derived from maternal exposure to HFO WAFs exhibited significantly higher levels when compared to those derived from the control and paternal exposure. The TAC level of maternal exposure also showed a significant increase with HFO WAFs’ concentrations. However, sperm and EF showed no significant difference with the control, respectively (Figure 3). The data for the TAC content are listed in Table S1.

### 3.3. Lipid Peroxidation Level

**Figure 4:**
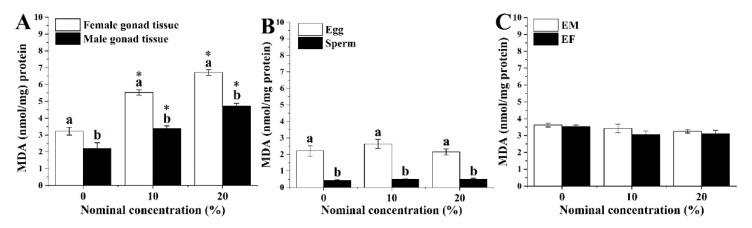
Change of lipid peroxidation (LPO) in (**A**) gonad tissues, (**B**) gametes and (**C**) larvae derived from different parental exposed behaviors with HFO WAFs for seven days. Data are presented as means ± SD. Lowercase letters indicate significant differences between sexes. An asterisk (*) labeled above bars indicates significant differences between treatments and the control (*p* < 0.05).

LPO levels in the gonad tissues, gametes and larvae of sea urchins were tested after exposure to HFO WAFs (10% and 20%). Following seven days of treatment, sea urchins exposed to different HFO WAFs could affect the LPO level (Figure 4A). The LPO levels in female gonad tissues were significantly greater (on average 1.5-fold higher) than in male gonad tissues. In contrast, gametes released from parental exposure maintained low levels of LPO, not differing from the control (Figure 4B). Sperms maintained a significantly lower level of LPO than eggs. However, there were no significant changes in the LPO levels of sea urchin larvae from EF and EM crosses (Figure 4C). The data on the LPO content are listed in Table S1.

### 3.4. Malformed Offspring

**Figure 5:**
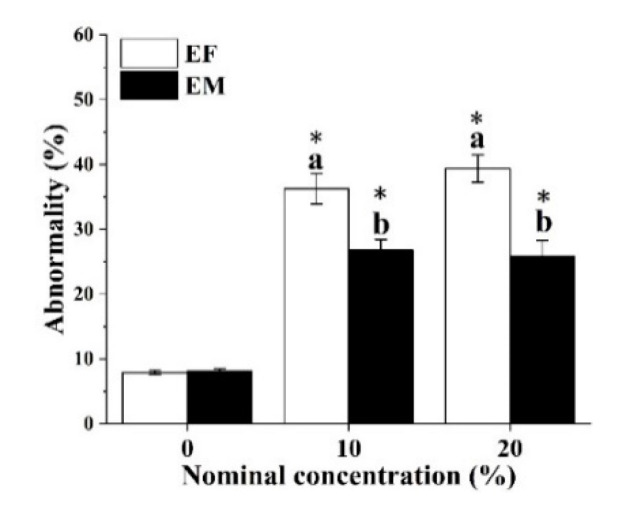
Morphological abnormalities of 48 hpf larvae derived from different parental exposed behaviors with HFO WAFs. Data were presented as means ± SD. Lowercase letters indicate significant differences between sexes. An asterisk (*) labeled above bars indicates significant differences between different treatments and the control (*p* < 0.05).

After being exposed to medium and higher concentrations, larvae derived from parental exposure showed a significantly increased (EF: 36.27 ± 2.35% at 10% and 39.35 ± 1.63% at 20%. EM: 26.8 ± 1.63% at 10% and 25.89 ± 2.43% at 20%) abnormality level in relation to the control (EF was 7.52 ± 0.32%, and EM was 8.05 ± 0.37%). Larvae derived from maternal exposure to HFO WAFs (10% and 20%) exhibited a significantly higher abnormality than those derived from paternal exposure (Figure 5).

## 4. Discussion

In this study, we separately treated sea urchin females and males with HFO WAF for seven days, in order to explore the sex-specific differences in the toxic effects of HFO WAF on sea urchins. To the best of our knowledge, few studies were focused on the sexual effect of the toxicity of oil pollution on sea urchins. Our results showed significant sex differences in the bioaccumulation of PAHs in sea urchins. In our study, the concentration of PAHs in the sea urchin female gonad tissues was higher than that in males, and sperms maintained a significantly lower concentration of PAHs than eggs. It is generally known that PAHs are highly lipid-soluble and could be easily accumulated in the lipid-rich tissues of marine organisms [28]. Moreover, previous findings revealed that sea urchin females had a higher lipid content in ovaries than the content in testes [5]. Therefore, the sex differences of PAHs bioaccumulation in sea urchin gonad tissues might be related to the differences in the lipid content of gonad tissues. In addition, this study found that PAHs concentrations in the gonad tissues of sea urchin females and males were both higher than those in the eggs and sperm. Previous studies found that parental sea urchins’ gonad tissues might protect their gametes against pollutant stress [29,30]. Therefore, the concentration of PAHs in sea urchin gonad tissues was higher than that in gametes, which might be related to sea urchins’ protection of their gametes. It is worth noting that HFO WAF exposure caused the bioaccumulation of PAHs in the gonad tissues and gametes of sea urchins, indicating that parental sea urchin exposure to HFO WAF might transfer PAHs from the gonad tissues to their gametes, which could further transfer to the offspring and might influence the fitness of sea urchin offspring; this, however, requires further investigation.

Numerous studies have reported that exposure to PAHs can lead to the overproduction of ROS in aquatic organisms [31,32]. The scavenging and production of ROS in vivo are a process of dynamic balance [11]. The scavenging of ROS is mainly accomplished through the antioxidant defense system [14]. Therefore, the increase of the ROS level will stimulate the increase of the antioxidant defense capacity in order to maintain the ROS level homeostasis in organisms. The rate of ROS production exceeding the antioxidant defense capacity might cause oxidative stress, manifesting as oxidative damage of macromolecules (DNA, protein and lipid) [11,22]. Thus, this study further evaluated the TAC and LPO levels of the gonad tissues (ovaries and testes), gametes (egg and sperm) and larvae of paternal sea urchins. We found that sea urchin exposed to HFO WAF had higher TAC levels in ovaries than in testes, which was consistent with other studies that reported that sea urchins’ ovaries had higher antioxidant (ascorbate) levels than testes had [33]. Moreover, our study showed that the sperms showed no significant differences in TAC levels, which might be associated with the reduction of sperms’ cytoplasm and might subsequently lead to a deficiency in the antioxidant defense [34]. These results indicated that sea urchin exposed to HFO WAF produced oxidative stress, which might cause oxidative damage in vivo. In this study, we found that larvae derived from maternal exposure to HFO WAF caused significantly higher TAC levels than those in the control, indicating that a maternally inherited protection might enhance the TAC level of larvae in order to minimize LPO [18]. In addition, after the organism is subjected to environmental stresses, the destruction of the body antioxidant defense system may cause LPO of polyunsaturated fatty acids (PUFA) and other lipid compounds in marine organisms exposed to environmental pressure [35]. Previous studies have reported that in sea urchins the ovaries had a higher lipid content than the testes and that the fatty acids composition in ovaries showed no significant difference to that in testes [5]. This indicated that the higher level of LPO in female gonad tissues than in males might be related to females with higher PUFA contents. Previous studies provided evidence that the PUFA content in eggs (56%) was higher than that in sperms (50.4%) [36]. This evidence might be one of the reasons that LPO in eggs were significantly higher than in sperms. However, further research is needed to explore the mechanisms that might explain the reasons for the differences in the lower level of LPO in sperm.

In this study, we found that both maternal and paternal sea urchin exposure to HFO WAF had adverse effects on their larval development. Maternal inheritance may have protected larvae and reduced the oxidative damage, but the morphological abnormalities of larvae were not reduced. The effect of HFO WAF on the development of sea urchin offspring may involve the trade-off of energy distribution among different functions in vivo. Previous studies reported that the energy resources of sea urchins were allocated so as to resist oxidative damage, and this caused a reduction in the supply to larva development, leading to an increase in the morphological abnormalities of larvae [18,22]. In addition, we found that paternal exposure showed no significant differences in the TAC and LPO levels, whereas the larvae’s morphological abnormalities still increased. Our previous studies found that the DNA damage level increased in sperms following paternal exposure to HFO [22], causing severe morphological abnormalities in their offspring [37]. Currently, previous studies have suggested that the life-history variation may be related to antioxidant parameters [38]. Therefore, it is largely uncertain whether the morphological abnormalities of larvae derived from parental sea urchins exposed to HFO WAFs is related to oxidative damage or not.

## 5. Conclusions

This study revealed sex-specific differences in the toxic effect of HFO WAFs on parental sea urchin gonad tissues, gametes and their offspring. Our results showed that sea urchin exposure to HFO WAF for seven days caused a PAHs bioaccumulation and TAC level increase in gonad tissues and gametes. PAHs bioaccumulation, TAC and LPO in the female and male gonad tissues, as well as in the eggs and sperms, were significantly different. Our results also showed that parental exposure to HFO WAF caused severe morphological abnormality in the offspring. However, there was insufficient proof to show transgenerational effects of HFO on sea urchins, when measurements were limited to TAC, LPO and their morphology. Overall, the results greatly expanded previous studies on each sex-specific difference and provided new insights into the toxic effects induced by HFO WAF on the offspring of sea urchin females and males. Serious damage to sea urchin exposure to HFO WAF is a potential threat to biodiversity and the survival of sea urchins. Going forward, further research should explore the transgenerational long-term effect of HFO on the offspring of sea urchins. In addition, in order to provide an effective assessment of oil spills, more studies using other benthic organisms are needed to expand the results of this study.

## Data Availability

Data sharing not applicable.

## Supplementary Materials

The following are available online at https://www.mdpi.com/1660-4601/18/2/499/s1, Table S1. TAC and LPO in sea urchin gonad tissues (female and male), gametes (egg and sperm) and offspring (EF and EM) exposed to HFO-WAFs. Values are means ± SD (n = 3); for gametes; n = 1 pooled sample of eggs or sperm from 6 sea urchins.
