# Supplementary material for: Sex-Specific Differences in the Toxic Effects of Heavy Fuel Oil on Sea Urchin (Strongylocentrotus intermedius)
Source: Int J Environ Res Public Health. 2021 Jan 9;18(2):499. doi: 10.3390/ijerph18020499 (PMC7827743; doi:10.3390/ijerph18020499)
Supplement: Supplementary file 1 [file ijerph-18-00499-s001.pdf]

International Journal of
Environmental Research and Public Health

MDPI

Article

Sex-Specific Differences in the Toxic Effects of Heavy Fuel Oil on Sea Urchin (*Strongylocentrotus intermedius*)

Xuanbo Wang, Hang Ren, Xishan Li, Huishu Chen, Zhonglei Ju and Deqi Xiong *

College of Environmental Science and Engineering, Dalian Maritime University, Dalian 116026, China; wangxuanbo@hotmail.com (X.W.); renhang960810@163.com (H.R.); lxsdmu@outlook.com (X.L.); 15942678549@163.com (H.C.); jzl621925@163.com (Z.J.); xiongdq@dlmu.edu.cn (D.X.)

* Correspondence: xiongdq@dlmu.edu.cn

Supplementary Materials

Table S1. TAC and LPO in sea urchin gonad tissues (female and male), gametes (egg and sperm) and offspring (EF and EM) exposed to HFO-WAFs. Values are means ± SD (n = 3); for gametes; n = 1 pooled sample of eggs or sperm from 6 sea urchins.

	**Total antioxidant capacity (U/mg protein)**			**Lipid peroxidation (nmol/mg protein)**		
	**Control**	**10%**	**20%**	**Control**	**10%**	**20%**
Female	2.2 ± 0.27	4.49 ± 0.57	4.6 ± 0.15	3.23 ± 0.23	5.53 ± 0.16	6.72 ± 0.17
Male	1.45 ± 0.27	2.87 ± 0.31	2.48 ± 0.08	2.19 ± 0.35	3.39 ± 0.15	4.72 ± 0.17
Egg	1.12 ± 0.2	1.69 ± 0.2	2.19 ± 0.35	3.39 ± 0.15	4.72 ± 0.17	2.19 ± 0.35
Sperm	0.2 ± 0.02	0.31 ±0.01	0.31 ± 0.02	0.42 ± 0.04	0.49 ± 0.02	0.49 ± 0.07
EF	2.9 ± 0.2	4.52 ± 0.24	5.28 ± 0.23	3.62 ± 0.11	3.42 ± 0.26	3.25 ± 0.1
EM	2.87 ± 0.21	3.11 ± 0.16	2.98 ± 0.22	3.54 ± 0.08	3.06 ± 0.22	3.11 ± 0.2
